# Prognosis-Predicting Model Based on [^18^F]fluorodeoxyglucose PET Metabolic Parameters in Locally Advanced Cervical Cancer Patients Treated with Concurrent Chemoradiotherapy: Multi-Center Retrospective Study

**DOI:** 10.3390/jcm9020427

**Published:** 2020-02-05

**Authors:** Won Kee Lee, Gun Oh Chong, Shin Young Jeong, Hyun Jung Lee, Shin-Hyung Park, Jung Min Ryu, Youn Seok Choi, Sungmin Kang, Yu-Jin Koo, Dae Hyung Lee, Eunjung Kong, Sang-Woo Lee

**Affiliations:** 1 Medical Research Collaboration Center in Kyungpook National University Hospital, Department of Medical Informatics, School of Medicine, Kyungpook National University, Daegu 41944, Korea; wonlee@knu.ac.kr; 2 Department of Obstetrics and Gynecology, Kyungpook National University Chilgok Hospital, Daegu 41404, Korea; 3 Department of Obstetrics and Gynecology, School of Medicine, Kyungpook National University, Daegu 41944, Korea; obgy1019@hanmail.net (H.J.L.); swleenm@knu.ac.kr (S.-W.L.); 4 Department of Nuclear Medicine, Kyungpook National University Chilgok Hospital, Daegu 41404, Korea; 5 Department of Nuclear Medicine, School of Medicine, Kyungpook National University, Daegu 41944, Korea; 6 Department of Obstetrics and Gynecology, Kyungpook National University Hospital, Daegu 41944, Korea; 7 Department of Radiation Oncology, Kyungpook National University Hospital, Daegu 41944, Korea; shinhyungpark@knu.ac.kr; 8 Department of Radiation Oncology, School of Medicine, Kyungpook National University, Daegu 41944, Korea; 9 Department of Obstetrics and Gynecology, School of medicine, Daegu Catholic University, Daegu 42472, Korea; medgirl87@naver.com (J.M.R.); drcys@cu.ac.kr (Y.S.C.); 10 Department of Nuclear Medicine, School of Medicine, Daegu Catholic University, Daegu 42472, Korea; kufa77@hanmail.net; 11 Department of Obstetrics and Gynecology, Yeungnam University Medical School and Hospital, Daegu 42415, Korea; yujinkoo@ynu.ac.kr (Y.-J.K.); leebhy@ynu.ac.kr (D.H.L.); 12 Department of Nuclear Medicine, Yeungnam University Medical School and Hospital, Daegu 42415, Korea; ejkong@ymc.yu.ac.kr

**Keywords:** risk model, nomogram, locally advanced cervical cancer, ^18^F-FDG PET/CT, concurrent chemoradiotherapy, prognosis

## Abstract

This study aimed to develop a prognosis-predicting model based on [^18^F]fluorodeoxyglucose positron emission tomography/computed tomography (^18^F-FDG PET/CT) and clinicopathologic factors in locally advanced cervical cancer patients treated with concurrent chemoradiotherapy (CCRT). The medical records of 270 locally advanced cervical cancer patients who were treated with CCRT were collected from three institutions and reviewed retrospectively. A nomogram was used for predicting 2-year disease-free survival (DFS) and 5-year overall survival (OS) based on Cox proportional hazards regression. Predictor variables included nodal maximum standardized uptake value (SUVmax), primary tumor SUVmax, age, tumor size, stage, serum squamous cell carcinoma antigen level, and human papillomavirus status. Internal nomogram validation was performed. A nomogram for predicting the 2-year DFS and 5-year OS was constructed using six and seven parameters, respectively. With a focus on 2-year DFS, our model found nodal SUVmax to be the highest weighted negative prognostic factor. With a focus on 5-year OS, young age was the highest weighted negative prognostic factor. The concordance index was 0.75 and 0.78 for the 2-year DFS and 5-year OS, respectively. This nomogram is a predictive tool that can be used to counsel patients for predicting survival outcomes. Moreover, our prognosis-predicting model may make it possible to personalize treatment.

## 1. Introduction

For locally advanced cervical cancer [International Federation of Gynecology and Obstetrics (FIGO) stage IIB to IV], concurrent chemoradiotherapy (CCRT) using a cisplatin-based regimen is currently the standard treatment modality [1,2]. The contribution of CCRT toward an improvement in survival outcomes in cervical cancer has been well confirmed, and a complete clinical response is achieved in 70%–90% patients [3,4]. However, about one-third of patients with cervical cancer experience recurrence, and most of the recurrences develop within 2 years after completion of therapy [5]. Unfortunately, there is no exact biomarker to predict tumor recurrence in locally advanced cervical cancer treated with CCRT. Therefore, accurate prediction of tumor recurrence may be helpful in improving survival outcomes with appropriate risk reduction.

Recently, several studies have focused on the development of biomarkers using [^18^F]fluorodeoxyglucose positron emission tomography/computed tomography (^18^F-FDG PET/CT) for the prediction of tumor recurrence in locally advanced cervical cancer [6,7,8,9,10]. Moreover, radiomics from diffusion-weighted imaging-magnetic resonance imaging and ^18^F-FDG PET/CT were independent predictors of recurrence in locally advanced cervical cancer treated with CCRT [11]. Previously, we have attempted to discover a biomarker for predicting tumor recurrence using pre-treatment ^18^F-FDG PET/CT in locally advanced cervical cancers treated with CCRT, and found only nodal maximum standardized uptake value (SUVmax) to be the most powerful biomarker for predicting tumor recurrence [12]. Moreover, we evaluated the prognostic value of intratumoral metabolic heterogeneity on ^18^F-FDG PET/CT, but it did not show superiority over traditional metabolic parameters [13]. 

Nomograms have been used to estimate oncological outcomes for locally advanced cervical cancers treated with CCRT [14,15,16,17,18]. However, only one study has reported ^18^F-FDG PET/CT-based prognostic nomograms for locally advanced cervical cancer [19]. In this study, only metabolic parameters were included as predictor variables for nomograms. Therefore, we hypothesized that risk models based on metabolic parameters on ^18^F-FDG PET/CT and clinicopathologic characteristics may be useful for predicting tumor recurrence or individualizing treatment.

The aim of the present study was to develop risk models for predicting tumor recurrence and survival in locally advanced cervical cancers treated with CCRT using metabolic parameters on ^18^F-FDG PET/CT and clinicopathologic parameters.

## 2. Materials and Methods

### 2.1. Patients

Three institutions participated in our retrospective study. The method of patient allocation was predetermined before analysis. For this study, we enrolled 270 patients with biopsy-confirmed cervical cancer treated with CCRT between November 2004 and November 2016 from three institutions (Kyungpook National University Chilgok Hospital, Daegu Catholic University Medical Center, and Yeungnam University Medical Center). Some of the enrolled patients at Kyungpook National University Chilgok Hospital included patients who had been included in previous studies [12]. Retrospective data collection and analysis were approved by the Institutional Review Boards of each institution. The need for informed consent was waived because of the retrospective design of the study. The patients were staged according to the FIGO staging system [20]. All patients had undergone ^18^F-FDG PET/CT for initial diagnosis, staging, and radiotherapy planning. Patients with a distant metastatic disease or history of previous surgery, or those who had undergone radiotherapy or chemotherapy, were excluded from the study.

Clinicopathological parameters, including age, serum squamous cell carcinoma (SCC) antigen, FIGO stage, histology, primary tumor size, hemoglobin and pre-treatment human papilloma virus (HPV) status, and presence of pelvic and paraaortic lymph node metastasis, were reviewed and retrieved. SUVmax of primary tumor and regional lymph node was measured by ^18^F-FDG PET/CT.

### 2.2. Treatment

All patients were treated with a combination of external beam radiotherapy (EBRT) and high-dose-rate (HDR) intracavitary brachytherapy (ICR) with curative intent. EBRT was delivered to the whole pelvis using a 3-dimensional conformal radiation therapy (3D-CRT) 4-field box technique (1.8 Gy daily fractions, five times a week, for a total dose of 45 Gy). Extended-field radiotherapy, including to the pelvis and paraaortic nodal area, was delivered for patients with paraaortic nodal involvement. A parametrial boost of 10 Gy in 5 fractions was additionally given to patients with parametrial involvement. HDR ICR initiated after delivery of an EBRT dose of 39.6 Gy. We used a revised definition of Point A that references the point “2 cm lateral to the center of the uterine canal and 2 cm superior from the cervical os [21]. For specifying rectal and bladder doses, the standard locations established by the International Commission for Radiation Units (ICRU) in Report 38 was used. The dose to the nominal rectal and bladder point was kept below 80% of the dose to Point A. Weekly cisplatin at a dose of 40 mg/m^2^ was administered during radiotherapy. The first course of cisplatin was administered on day 1 of radiotherapy. For calibration of HDR Ir-192 sources, the Nucletron Source Dosimetry System (Nucletron B.V., Veenendaal, The Netherlands) was used, which was composed of Physikalisch-Technische Werkstatten (PTW) (Model No. 077092) well-type ionization chamber and Unidos Webline electrometer.

### 2.3. Pre-Treatment Assessment

Tumor size was measured by magnetic resonance imaging. If tumor size was reported in three axes, the largest diameter was considered to be the tumor size. 

All ^18^F-FDG PET/CTs were performed with dedicated PET/CT scanners (Discovery STe, GE Healthcare at Kyungpook National University Chilgok Hospital and Daegu Catholic University School of Medicine, Discovery ST/Discovery VCT, GE Healthcare at Yeungnam University Medical Center). All patients fasted for at least 6 h before the PET/CT. Blood glucose levels were measured and were required to be less than 150 mg/dL. A dose of approximately 5.5 MBq/kg of FDG was intravenously administered. At all institutions, PET/CT was performed from the thigh to the head, about 60 min after intravenous administration of FDG. Whole-body CT was performed without contrast enhancement using the following standard protocols: 140 kVp, 30 to 170 mAs adjusted to the patient’s body weight, and 3-mm slice thickness (Discovery STe); 100–120 kVp, 100–120 mAs, and 3.75-mm slice thickness (Discovery ST and Discovery VCT). An emission scan was performed in 3D mode after the CT scan. The acquisition time was 3 min per bed position. PET images were reconstructed by an iterative ordered subset expectation maximization algorithm using CT images for attenuation correction (Discovery STe, 4 iterations, 8 subsets; Discovery ST/Discovery VCT, 2 iterations, 8 subsets).

### 2.4. Image Analysis

All FDG PET/CT imaging data were transferred to the image archive server (Kyungpook National University Chilgok Hospital, Korea) using the Digital Imaging and Communications in Medicine standard. All images were centrally reviewed by two nuclear medicine physicians using the volume viewer software on an Advantage Workstation 4.5 (GE Medical Systems, Milwaukee, WI, USA), which provides a convenient and automatic method to delineate the volume of interest using an isocontour threshold method based on SUV. For semiquantitative analysis, SUVmax was designated as the highest SUV of the primary tumor and regional lymph nodes. SUVmax was obtained using the following formula: *SUVmax = maximum activity in the region of interest (MBq/g)/(injected dose [MBq]/body weight [g])*. 

### 2.5. Clinical Follow-up

Clinical follow-ups of patients were performed every 3 months for 2 years, every 6 months between 2 and 5 years, and annually thereafter. Failure was defined as biopsy-proven recurrence or documentation of disease progression on serial imaging studies. 

### 2.6. Statistical Analysis

The number of recurrent patients and the recurrence rate were calculated for each independent variable level. The Contal and O’Quigley technique was used to select the cutoff value for each continuous independent variable. The estimate of 2-year disease-free survival DFS, 5-year overall survival (OS) and 95% confidence interval (CI) for each independent variable level was determined using the Kaplan–Meier estimator, and the statistical significance was tested using the log-rank test for each independent variable. The crude proportional hazard rate (HR) of 2-year DFS and 5-year OS for each independent variable was estimated using the univariate and multivariate Cox proportional-hazards model (PHM) for the adjusted HR. 

To calculate conditional survival probability intuitively, nomogram of the 2-year DFS and 5-year OS prediction models based on a cox’s PHM was used, as described previously [22]. To find out whether the risk score of nomogram using independent variables including FDG PET metabolic parameters was predictive of cervical cancer recurrence, we classified them into high or low risk groups using the Contal and O’ Quigley technique and compared survival functions. In addition, the concordance index of the nomogram was calculated from the original data set, and the standard error for the 95% confidence interval was obtained by the bootstrap method.

Survival analyses was performed using SAS version 9.4 (SAS Institute Inc., Cary, NC) and the figure plotting was performed using the rms package of R version 3.5.2 for windows. Data were determined to be statistically significant if the *p*-value was less than 0.05.

### 2.7. Validation of the Nomogram

The nomogram’s predictive accuracy was measured by the concordance index, which quantifies the level of concordance between predicted probabilities and the actual chance of recurrence or death. This was calculated by bootstrapping the samples from the original 270 patients used to fit the Cox model and served as an unbiased measure of the ability of the nomogram to discriminate patients. The nomogram was calibrated by grouping patients with respect to their nomogram-predicted probabilities and comparing group means with observed Kaplan–Meier estimates for DFS and OS. Bootstrapping was then repeated 200 times.

## 3. Results

Clinicopathological and PET metabolic parameters of the model derivation cohort. The characteristics of the 270 enrolled patients are summarized in Table 1 and Table 2. The median follow-up period was 49.5 months (range, 3–125 months). During the follow-up period, 69 patients (25.6%) had a recurrence and 33 patients (12.2%) died of disease. 

### 3.1. Independent Prognostic Factors for the Risk Model

Multivariate Cox proportional-hazards model was used to evaluate independent prognostic factors and estimate their effect on DFS and OS. Six variables were identified as independent risk factors for DFS; these included age, FIGO stage, tumor size, serum SCC antigen levels, pSUVmax, and nSUVmax (Table 3). Variables for OS included HPV status in addition to the previous six variables (Table 4).

### 3.2. Nomogram and Computation of Risk Scores for Predicting Tumor Recurrence

**Figure 1:**
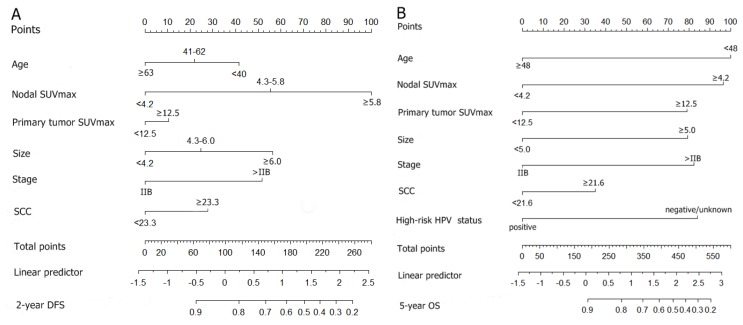
Nomogram for predicting the 2-year disease-free survival (**A**) and 5-year overall survival (**B**). Instructions for use of the nomogram. Draw a vertical line on the corresponding axis of each variable to the top line labeled “Points” to calculate the score for each variable. Add the number of points for all variables then draw a vertical line from the axis labeled “Total points” until it intercepts each of the survival axes to determine the 2-year disease-free survival and 5-year overall survival probability.

A nomogram was constructed based on these six and seven independent risk factors for 2-year DFS and 5-year OS, respectively. In the nomogram, the point of each risk factor was identified on the top scale, and the total points were calculated by totaling the points of the risk factors. The probability of mortality can be assigned by applying the total points to the bottom scale of nomogram (Figure 1). The total prognostic score on the bottom axes indicates the probability of 2-year DFS and 5-year OS.

### 3.3. Internal Validation

**Figure 2:**
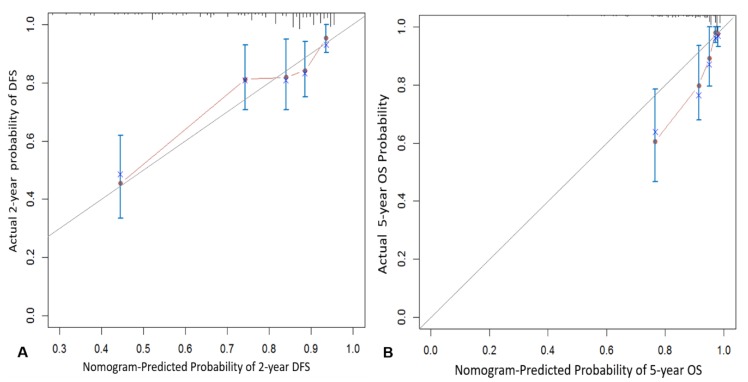
The calibration curve for predicting the 2-year disease-free survival (**A**) and 5-year overall survival (**B**).

The calibration plots for the probability of 2-year DFS and 5-year OS demonstrated optimal agreement between prediction by the nomogram and actual observation. The calibration curve and concordance for the nomogram are illustrated in Figure 2. Based on the nomogram, the concordance index was 0.75 (95% CI, 0.69–0.81) for 2-year DFS and 0.78 (95% CI, 0.71–0.85) for 5-year OS.

### 3.4. Discrimination Ability of Nomogram for Prognosis

**Figure 3:**
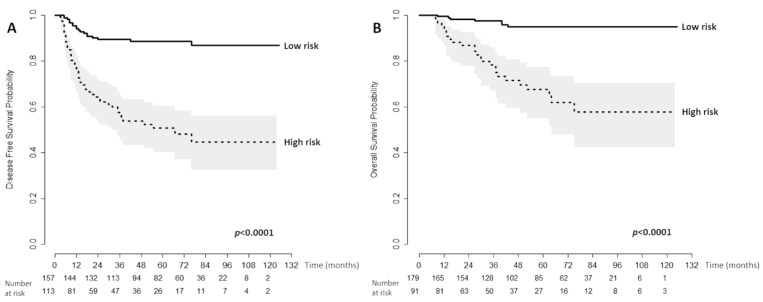
Kaplan–Meier disease-free survival (**A**) and overall survival curves (**B**) for high-risk and low-risk groups.

The discrimination ability of the nomogram in predicting DFS and OS was analyzed by dividing the predicted probabilities of survival outcomes in two groups that were then used to plot the Kaplan–Meier curve. Optimal cutoff values were determined by an algorithm for the maximization of hazard ratio to divide the high- and low-risk groups. The nomogram could stratify patients into low- and high-risk subgroups, and cut-off scores for DFS and OS were 102 and 214, respectively. Furthermore, DFS and OS were significantly higher in the low-risk group than in the high-risk group (*p* < 0.0001, Figure 3).

## 4. Discussion

In this study, we developed and validated a nomogram based on the ^18^F-FDG PET /CT metabolic and clinicopathological parameters for predicting 2-year DFS and 5-year OS. The regression model used as many independent variables as possible to make the model predictive. The variance inflation factors (VIFs) between the independent variables were all less than 1.2, and there were no multiple collinearity problems. 

A nomogram is a predictive tool that generates the numerical probability of a clinical event by creating a simple graphical representation of a statistical predictive model [22]. Several prognostic models have been developed to predict mortality, cause-specific mortality, disease recurrence, and distant or paraaortic recurrence over a specific duration in patients diagnosed with locally advanced cervical cancer treated with CCRT [14,15,16,17,18,19,23]. Various types of prognostic factors—such as age, race, FIGO stage, histological type, degree of differentiation, lymph node involvement and location, tumor volume, SUVmax of cervical tumor, serum SCC antigen levels, and treatment received—have been used as components of the prognostic predictive models [24]. In our study, age, FIGO stage, tumor size, serum SCC antigen levels, pSUVmax, nSUVmax, and HPV status were the included prognostic factors for predicting 2-year DFS and 5-year OS. Most previous studies only applied clinicopathological parameters to prognostic nomogram [14,15,16,17,18]. Only one study applied metabolic parameters obtained from ^18^F-FDG PET/CT to the prognostic nomogram for locally advanced cervical cancer patients treated with CCRT [19]. In this previous study, pretreatment ^18^F-FDG PET lymph node status, cervical tumor SUVmax, and PET tumor volume combined in a nomogram created a good model for predicting recurrence-free survival and OS [19]. 

Previously, we evaluated the ^18^F-FDG PET/CT metabolic parameters for predicting tumor recurrence in locally advanced cervical cancer treated with CCRT. Among SUVmax, metabolic tumor volume, total lesion glycolysis of the primary tumor, regional lymph node, and whole body, and tumor heterogeneity, the SUVmax of regional lymph node was the most powerful biomarker for predicting tumor recurrence in locally advanced cervical cancer treated with CCRT [12,13]. Previous ^18^F-FDG PET-based prognostic nomograms included lymph node status as a prognostic factor; however, only lymph node level was included [19]. Moreover, clinicopathological parameters were not used for developing prognostic nomogams [17]. In this study, the quantitative metabolic parameter for regional lymph nodes, i.e., nodal SUVmax, and clinicopathological parameters were used to develop a prognostic risk model using nomograms. With a focus on 2-year DFS, our model found nodal SUVmax to be the highest-weighted negative prognostic factor, which corresponded with the findings of our previous studies [12,13]. With a focus on 5-year OS, young age was the highest-weighted negative prognostic factor. Young age was reported as one of the poor prognostic factors [25,26,27]. Compared to older patients, younger cervical cancer patients often exhibit the following characteristics: immune deficiency; tobacco smoking; high serum hormone levels; cervical erosions; HPV16 infection; and high levels of survivin, cyclooxygenase 2, matrix metalloproteinase and CD44 expression. In addition, younger patients with cervical cancer also exhibit poor tumor differentiation and lymph node metastasis. Our nomograms, especially for 2-year DFS and 5-year OS, showed good prediction accuracies, as indicated by the concordance index of 0.75 and 0.78, respectively. A concordance index greater than 0.7 suggests that the 2-year DFS and 5-year OS outcomes are well modeled by nomograms based on ^18^F-FDG PET/CT metabolic parameters and clinicopathological parameters [28]. 

Our study has some noteworthy limitations. First, it is a retrospective study. The possibility of selection bias exists. Because this study was conducted only on patients who underwent ^18^F-FDG PET, there may be sampling bias. Second, three different PET/CT scanners were used. Thus, there may have been some differences between institutions with regard to image acquisition and reconstruction. To minimize these differences, reconstruction processing and imaging analyses were performed at a single institution (KNUCH) using the same software (Advantage Workstation 4.5 software) for all calculations. Third, external validation was not performed.

Despites these limitations, our study offers some unique and significant findings and it differs from previous studies in several aspects. This study is the largest study so far, to develop a prognostic nomogram based on pretreatment ^18^F-FDG PET/CT metabolic and clinicopathologic factors with 270 cases of locally advanced cervical cancer treated with CCRT. Moreover, our prognosis-predicting model is the first nomogram that combines ^18^F-FDG PET/CT metabolic and clinicopathological parameters. 

## 5. Conclusion

In conclusion, we developed and internally validated a risk assessment model using a nomogram for predicting the probability of 2-year DFS and 5-year OS after CCRT in locally advanced cervical cancer. This nomogram may be helpful in identifying patients with high risk of recurrence or compromised survival and allow physicians to choose appropriate adjuvant treatments, such as hysterectomy or chemotherapy, and counsel patients. Moreover, our prognostic variables offered by a nomogram based on ^18^F-FDG PET/CT and clinicopathological characteristics could be used for the homogenization of various prognostic nomograms.

## Figures and Tables

**Table 1:** Clinicopathologic and [^18^F]fluorodeoxyglucose PET metabolic parameters of the model derivation cohort for 2-year disease-free survival.

Variables		Sample Size (n)	Recurrence	Log-Rank Test		
			(n, %)	2-Year DF (95% CI)	χ^2^	*p*
Age	<40	34	12 (35.3)	0.66 (0.49–0.82)	4.13	0.127
	41–62	167	45 (26.6)	0.79 (0.73–0.85)		
	≥63	69	12 (17.4)	0.84 (0.75–0.93)		
FIGO stage	IIB	215	45 (20.9)	0.83 (0.77–0.88)	15.84	<0.001
	≥IIB	55	24 (43.6)	0.63 (0.49–0.76)		
Histology	SCC	243	60 (24.7)	0.79 (0.74–0.85)	1.33	0.249
	AC/ASC	27	9 (33.3)	0.71 (0.53–0.90)		
Size	<4.2	59	7 (11.9)	0.95 (0.89–1.01)	14.34	0.001
	4.3–6.0	66	13 (19.7)	0.86 (0.77–0.94)		
	≥6.0	145	49 (33.8)	0.69 (0.61–0.77)		
SCC antigen	<23.3	223	48 (21.5)	0.82 (0.77–0.87)	21.74	<0.001
	≥23.3	47	21 (44.7)	0.63 (0.49–0.77)		
Hemoglobin	<12	150	45 (30.0)	0.74 (0.67–0.81)	3.92	0.048
	≥12	120	24 (20.0)	0.84 (0.78–0.91)		
HPV status	Positive	160	38 (23.8)	0.81 (0.74–0.87)	1.02	0.312
	Negative/Unknown	110	31 (28.2)	0.76 (0.67–0.84)		
pSUVmax	<12.5	148	27 (18.2)	0.88 (0.82–0.93)	10.04	0.002
	≥12.5	122	42 (34.4)	0.68 (0.60–0.76)		
nSUVmax	4.2	195	30 (15.4)	0.86 (0.81–0.91)	49.06	<0.001
	4.3–5.8	24	10 (41.7)	0.76 (0.57–0.94)		
	≥5.8	51	29 (56.9)	0.53 (0.39–0.67)		

DFS = disease-free survival; CI = confidence interval; FIGO = International Federation of Gynecology and Obstetrics; SCC = squamous cell carcinoma; AC = adenocarcinoma; ASC = adenosquamous cell carcinoma, HPV = human papillomavirus; pSUVmax = primary tumor maximum standardized uptake value; nSUVmax = nodal maximum standardized uptake value.

**Table 2:** Clinicopathologic and [^18^F]fluorodeoxyglucose PET metabolic parameters of the model derivation cohort for 5-year overall survival.

Variables		Sample Size (n)	Death	Log-Rank Test		
			(n, %)	5-Year OS (95% CI)	χ^2^	*p*
Age	<48	87	18 (20.7)	0.80 (0.70–0.89)	6.67	0.01
	≥48	183	15 (8.2)	0.89 (0.84–0.95)		
FIGO stage	IIB	215	22 (10.2)	0.88 (0.83–0.93)	6.49	0.011
	>IIB	55	11 (20.0)	0.75 (0.62–0.89)		
Histology	SCC	243	27 (11.1)	0.87 (0.82–0.92)	2.97	0.085
	AC/ASC	27	6 (22.2)	0.75 (0.55–0.95)		
Size	<5.0	165	13 (7.9)	0.93 (0.89–0.97)	11.86	0.001
	≥5.0	105	20 (19.0)	0.73 (0.62–0.83)		
SCC antigen	<21.6	217	20 (9.2)	0.90 (0.86–0.94)	10.36	0.001
	≥21.6	53	13 (24.5)	0.68 (0.53–0.83)		
Hemoglobin	<11.7	129	20 (15.5)	0.81 (0.73–0.89)	3.31	0.069
	≥11.7	141	13 (9.2)	0.90 (0.84–0.95)		
HPV status	Positive	160	13 (8.1)	0.89 (0.84–0.95)	5.9	0.015
	Negative/Unknown	110	20 (18.2)	0.81 (0.73–0.90)		
pSUVmax	<12.5	148	10 (6.8)	0.92 (0.87–0.97)	9.67	0.002
	≥12.5	122	23 (18.9)	0.79 (0.71–0.87)		
nSUVmax	<4.2	195	16 (8.2)	0.90 (0.86–0.95)	12.86	<0.001
	≥4.2	75	17 (22.7)	0.73 (0.60–0.86)		

OS = overall survival; CI = confidence interval; FIGO = International Federation of Gynecology and Obstetrics; SCC = squamous cell carcinoma; AC = adenocarcinoma; ASC = adenosquamous cell carcinoma, HPV = human papillomavirus; pSUVmax = primary tumor maximum standardized uptake value; nSUVmax = nodal maximum standardized uptake value.

**Table 3:** Hazard ratio by Cox’s proportional hazards model for risk factors of recurrence.

Variables		Univariate		Multivariate	
		HR (95% CI)	*p*	HR (95% CI)	*p*
Age	<40	2.26 (1.01–5.02)	0.047	1.79 (0.79–4.07)	0.164
	41–62	1.54 (0.82–2.91)	0.184	1.36 (0.70–2.64)	0.36
	≥63	1		1	
FIGO stage	IIB	1		1	
	>IIB	2.63 (1.60–4.33)	<0.001	2.07 (1.21–3.52)	0.008
Histology	AC	1			
	AC/ASC	1.53 (0.75–3.03)	0.255		
Tumor size	<4.2	1		1	
	4.3–6.0	1.81 (0.72–4.55)	0.205	1.42 (0.56–3.61)	0.468
	≥6.0	3.60 (1.63–7.95)	0.002	2.21 (0.97–5.04)	0.059
SCC antigen	<23.3	1		1	
	≥23.3	2.46 (1.47–4.12)	0.001	1.48 (0.87–2.52)	0.151
Hemoglobin	<12	1.64 (1.00–2.69)	0.051		
	≥12	1			
HPV status	Positive	1			
	Negative/Unknown	1.28 (0.79–2.05)	0.315		
pSUVmax	<12.5	1		1	
	≥12.5	2.14 (1.32–3.47)	0.002	1.16 (0.68–1.98)	0.59
nSUVmax	4.2	1		1	
	4.3–5.8	2.28 (1.77–2.94)	<0.001	2.18 (1.04–4.58)	0.04
	≥5.8			4.08 (2.35–7.08)	<0.001

HR = hazard ratio; CI = confidence interval; FIGO = International Federation of Gynecology and Obstetrics; SCC = squamous cell carcinoma; AC = adenocarcinoma; ASC = adenosquamous cell carcinoma, HPV = human papillomavirus; pSUVmax = primary tumor maximum standardized uptake value; nSUVmax = nodal maximum standardized uptake.

**Table 4:** Hazard ratio by Cox’s proportional hazards model for risk factors of death.

Variables	Level	Univariate		Multivariate	
		HR (95% CI)	*p*	HR (95% CI)	*p*
Age	<48	2.40 (1.21–4.76)	0.013	2.07 (1.02–4.22)	0.045
	≥48	1		1	
FIGO stage	IIB	1		1	
	>IIB	2.49 (1.48–6.03)	0.014	1.96 (0.92–4.16)	0.082
Histology	AC	1			
	AC/ASC	2.14 (0.88–5.17)	0.093		
Tumor size	<5.0	1		1	
	≥5.0	3.22 (1.59–6.50)	0.001	1.81 (0.88–3.74)	0.11
SCC antigen	<21.6	1		1	
	≥21.6	2.99 (1.48–6.03)	0.002	1.33 (0.62–2.85)	0.47
Hemoglobin	<11.7	1.89 (0.94–3.80)	0.051		
	≥11.7	1			
HPV status	Positive	1		1	
	Negative/Unknown	2.32 (1.15–4.66)	0.019	1.81 (0.88–3.74)	0.11
pSUVmax	<12.5	1		1	
	≥12.5	3.06 (1.46–6.44)	0.003	1.82 (0.83–3.97)	0.134
nSUVmax	<4.2	1		1	
	≥4.2	3.23 (1.64–6.46)	0.001	1.57 (1.07–2.31)	0.02

HR = hazard ratio; CI = confidence interval; FIGO = International Federation of Gynecology and Obstetrics; SCC = squamous cell carcinoma; AC = adenocarcinoma; ASC = adenosquamous cell carcinoma, HPV = human papillomavirus; pSUVmax = primary tumor maximum standardized uptake value; nSUVmax = nodal maximum standardized uptake.
